# Application of Antioxidant Mixture Prevents Cutaneous “Oxinflammaging” in Subjects Exposed to Particulate Matter

**DOI:** 10.1111/jocd.70306

**Published:** 2025-07-11

**Authors:** John Ivarsson, Xi Yan, Anna Guiotto, Alessandra Pecorelli, Mariáurea Matias Sarandy, Stephen Lynch, Sara Anderias, Hina Choudhary, Stacy White, Francesca Ferrara, Giuseppe Valacchi

**Affiliations:** ^1^ Department of Food, Bioprocessing and Nutrition Sciences Plants for Human Health Institute, NC Research Campus, NC State University Kannapolis North Carolina USA; ^2^ Department of Animal Sciences Plants for Human Health Institute, NC Research Campus, NC State University Kannapolis North Carolina USA; ^3^ L'Oréal Research and Innovation Clark New Jersey USA; ^4^ Department of Environmental Sciences and Prevention University of Ferrara Ferrara Italy; ^5^ SkinCeuticals New York New York USA; ^6^ Department of Chemical, Pharmaceuticals and Agricultural Sciences University of Ferrara Ferrara Italy; ^7^ Department of Food and Nutrition Kyung Hee University Seoul South Korea

**Keywords:** air pollution, OxInflammation, skin aging, skin barrier, topical application

## Abstract

**Background:**

Premature skin aging and the growing incidence of skin conditions are closely linked to daily exposure to environmental agents. Among all air pollutants, particulate matter (PM) is regarded as one of the most aggressive in terms of skin damage, promoting degradation of extracellular matrix (ECM) components and depletion of the cutaneous antioxidant defense via initiation of oxinflammatory reactions. Moreover, PM can penetrate damaged skin, exacerbating the effect of other pollutants and worsening the symptoms of existing skin conditions. Topical application of antioxidant compounds can limit and, in certain cases, even prevent skin damage.

**Objective:**

In the present study, a randomized clinical study was conducted to evaluate the protective effects of a topically applied antioxidant serum containing 15% ascorbic acid, 0.5% ferulic acid, and 1% tocopherol (AOX Mix) against the PM‐induced skin damage.

**Methods:**

For this purpose, the back of 15 female volunteers, between 19–40 years and with skin phototypes III and IV, was pre‐treated with AOX Mix and exposed daily to PM for 4 days. Skin sample biopsies were subsequently collected and subjected to histological analysis to evaluate the key markers related to skin “oxinflammaging”.

**Results:**

The AOX Mix demonstrated protective effects against the cutaneous degradation of ECM components, including type I and III collagens, elastin, and tropoelastin. It also mitigated markers of inflammation (COX2), oxidative stress (4‐hydroxynonenal), and structural damage (involucrin, filaggrin, claudin‐1, desmocollin 1).

**Conclusion:**

Regular topical application of an antioxidant cosmeceutical formulation represents an effective strategy to counteract the detrimental effects of environmental insults, particularly with respect to inflammaging.

## 1 Introduction

Environmental pollutants are major factors involved in the development of premature skin aging [1]. While ultraviolet radiation has long been considered the primary external agent responsible for skin damage, the growing release of man‐made toxin agents within the atmosphere—such as diesel exhaust from cars, volatile organic compounds (VOCs), nitric oxide (NO), cigarette smoke (CS), ozone (O_3_)—has been associated with a marked increase in skin pathologies including cancer and extrinsic skin aging [2, 3]. There are almost 8500 papers in PubMed related to the effects of air pollution on cutaneous tissues and the ability of pollution exposure to induce and exacerbate pro‐inflammatory skin conditions [2, 3].

Air pollutants have been shown to affect skin homeostasis by promoting oxidative and inflammatory responses (oxinflammation) that propagate throughout the cutaneous layers, affecting various skin components including proteins and lipids essential for maintaining skin integrity and barrier function [4, 5]. Particulate matter (PM) is one of the major noxious pollutants present in the air, composed of particles ranging in size from 0.1–10 μm, which can come in contact with multiple tissues, especially the lungs and skin. Deposition of PM on the skin's surface leads to the release of toxic substances such as transition metals and organic compounds, triggering the activation of transcription factors (i.e., aryl hydrocarbon receptor (AhR), nuclear factor‐b NF‐kB), that regulate cutaneous antioxidant and inflammatory responses [6, 7, 8]. All these reactions promote the establishment of an oxinflammatory environment that contributes to cutaneous barrier dysfunction, exacerbation of skin conditions, and premature aging [6, 9, 10].

Moreover, a recent study demonstrated that, similar to sun radiation, exposure to diesel exhaust particles (DEP) can induce an upregulation in cutaneous melanin levels via a redox‐regulated mechanism, promoting skin pigmentation. Topical application of an antioxidant mixture could prevent melanin production in response to DEP, suggesting that human skin produces melanin as a protective strategy to counteract the air pollution‐induced oxidative stress damage [11].

Although the penetration of PM into the skin layers is still under debate, some studies have shown that damaged skin is more prone to the infiltration of polycyclic aromatic hydrocarbons (PAHs), the major components of PM_2.5_ [12, 13], thereby exacerbating skin damage. Recently, multimodal nonlinear optical (MNLO) imaging revealed that while PM_2.5_ tends to accumulate in the apical layers of healthy skin, mostly in the *stratum corneum*, it can easily infiltrate the epidermis and dermis of damaged skin, exacerbating the release of inflammatory cytokines and interleukins such as IL‐1α and IL‐1β [14]. Considering that PM can promote skin barrier damage by altering the main components of the cornified cell envelope, such as filaggrin and keratins [6, 15], the skin might become more prone to the infiltration of other PM particles and more susceptible to the detrimental effects of other environmental insults, leading to exacerbated cutaneous damage.

Furthermore, PM can interact with other environmental pollutants, enhancing its reactivity and explicating a synergistic detrimental effect [13, 16]. For instance, the presence of UV has been found to exacerbate PM‐induced skin damage by activating mitogen‐activated protein kinase (MAPK) and AhR pathways, disrupting skin barrier proteins, increasing inflammation (IL‐1β), and inducing senescence [17]. Long‐term exposure to environmental pollutants contributes to the depletion of micronutrients and vitamins naturally present in the skin, as well as decreased levels of cutaneous antioxidant enzymes (i.e., catalase, superoxide dismutase, glutathione peroxidase etc.) that are essential for counteracting the air pollutants‐induced reactive oxygen species (ROS) production [18]. Considering the close interplay between the oxidative and inflammatory responses within cells [19], the loss of the cutaneous antioxidant defenses may compromise the resolution of inflammation promoted by air pollutants, exacerbating skin inflammaging.

For these reasons, topical application of antioxidant molecules has been identified as an effective strategy to prevent the establishment of an oxinflammatory imbalance promoted by air pollutants by reducing the production of ROS and/or replenishing the skin's natural antioxidant components, thereby maintaining skin homeostasis [20, 21, 22, 23].

In the present study, we investigated the protective effect of a topical antioxidant cosmeceutical mixture (AOX Mix) containing 15% ascorbic acid, 0.5% ferulic acid, and 1% tocopherol (CE Ferulic, SkinCeuticals Inc., New York, NY), in 15 healthy subjects exposed to PM. The inclusion of ferulic acid in cosmeceutical formulations has been shown to stabilize ascorbic acid and tocopherol, enhancing their skin absorption and antioxidant activity [24]. Female volunteers aged between 19–40 years, with skin phototypes III and IV, were screened and enrolled in the study. The back of the volunteers was pre‐treated with the AOX Mix and exposed to PM every day for 4 days, and then skin biopsies were analyzed for markers related to skin structure (filaggrin, involucrin, claudin‐1, DSC1), inflammation (cyclooxygenase 2, COX2), oxidative stress (4‐hydroxynonenal, 4HNE) and premature aging (type I and III collagens, elastin, tropoelastin depletion). Results showed that AOX Mix effectively prevented skin oxinflammatory responses and structural damage promoted by PM exposure.

## 2 Materials and Methods

### 2.1 Enrollment of Patients

After the approval of the Institutional Review Board (Independent Ethics Committee; Study Code 2122CMCL061; January 28, 2022), 15 female volunteers between 19–40 years old and with phototypes III and IV were screened and enrolled in the study based on the inclusion and exclusion criteria (presence of dermatological or systemic disorders, allergies, and medical conditions). The subjects avoided excessive sun exposure and the use of any other topical products for the entire duration of the study.

### 2.2 PM Exposure System and AOX Mix Application

For the study, the backs of the 15 volunteers were divided into different zones with a diameter of 6 cm for a total area of 28.27cm^2^ on the upper back:

Zone 1: Untreated and unexposed.

Zone 2: Untreated and exposed to particulate matter.

Zone 3: Treated with AOX mix and exposed to particulate matter.

As for the AOX mix, 2 mg/cm^2^ (2 drops) of the product were applied on the defined Zone 3 using a finger cot, 15–30 min after product application, Zone 2 and Zone 3 were exposed to 100 μg/m^3^ of particulate matter usually present in an urban area (NIST SRM 1648a) by the controlled pollution exposure system at a flow of 500 mL/min for 2 h.

To assess the exposure of patients, a Controlled Pollution Exposure System (CPES) (CH Technologies USA) was employed [25] that is based on a Vilnus Aerosol Generator (VAG) (CH Technologies USA) able to generate a mixture of particles and air released via the inlet of the exposure cup fixed on the subject's back. The rate flow of the air through the cup was set at 0.5 L/min and the concentration of the particles reaching the subject's skin, as well as the particle size distribution (PM1, PM2.5, PM10 and others), were analyzed and displayed by a FIDAS particle monitor (CH Technologies USA).

On the day of the exposure, the back of the participants was left uncovered for 15 min and then 3 randomly selected zones were defined and either treated with AOX Mix (15% ascorbic acid, 0.5% ferulic acid, and 1% tocopherol; CE Ferulic, SkinCeuticals Inc., New York, NY) for 30 min, then exposed or not to PM for 2 h, or left untreated (negative control, NT). The treatments were performed once daily for 4 days on the respective zone.

### 2.3 Collection of Human Skin Tissues

After 4 days of treatment, 3‐mm size punch biopsies were collected from the subject back's treated zones. Briefly, the patients were subjected to local anesthesia via an intradermal injection, and the treated skin area was disinfected. The punch biopsy was placed on the skin and moved down to the hypodermis by rotating 90°, using minimal pressure. After collection, the skin biopsies were placed in tubes filled with a preservative solution until the subsequent analysis.

### 2.4 Paraffin Embedding and Hematoxylin & Eosin (H&E) Staining of Skin Samples

Skin samples were transferred in a 10% neutral‐buffered formalin fixative solution for 24 h at RT, then subjected to dehydration in a series of alcohols (70%, 80%,90%,100%) and xylene.

Dehydrated samples were then embedded in paraffin and 4 μm thick tissue sections were cut with a microtome for further analysis. For hematoxylin and eosin (H&E) staining, tissue sections were rehydrated and stained with Mayer's hematoxylin solution (MHS32, Sigma‐Aldrich, St. Louis, MO, USA) for 7 min, then washed in tap water and stained with and aqueous eosin y solution (HT110216, Sigma‐Aldrich, St. Louis, MO, USA) for 1 min as previously described [16].

### 2.5 Immunofluorescence Staining of Tissue Sections

Immunofluorescence staining assay was performed as previously described [26]. The primary antibodies used for the overnight incubation at 4°C were suspended in PBS‐BSA 0.25% at the indicated dilutions (Table 1). Skin sections were then incubated for 1 h at RT with fluorochrome‐conjugated secondary antibodies (Alexa Fluor 568 and Alexa Fluor 488, Thermo Fisher Scientific Inc.) diluted 1:500 in 0.25% BSA in PBS.

**TABLE 1:** Antibodies specifications.

Antibody	Dilution	Catalog number and company
4HNE	1:400	AB5605, MilliporeSigma, Burlington, MA, USA
Filaggrin	1.50	sc‐66 192, Santa Cruz Biotechnology Inc., Dallas, TX, USA
Involucrin	1:50	sc‐21 748, Santa Cruz Biotechnology Inc.
COX2	1:400	NB100‐868, Novus Biologicals LLC, Centennial, CO, USA
Type I Collagen	1:200	EPR7785, Abcam, Waltham, MA, USA
Type III Collagen	1:50	Sc‐514 601 SantaCruz
Claudin‐1	1:50	sc‐166 338, Santa Cruz Biotechnology Inc., USA
Desmocollin 1	1:50	sc‐398 590, Santa Cruz Biotechnology Inc., USA
Elastin	1:200	AB9519, Abcam, Waltham, MA, USA
Tropoelastin	1:200	MBS606788, MyBioSource, USA

A DAPI solution (D1306, Thermo Fisher Scientific Inc.) was used to stain nuclei by incubating the tissue sections for 5 min at RT, and then slides were mounted onto glass slides using a fluorescence preservative mounting media (PermaFluor, TA006FM, ThermoFisher Scientific Inc.) A Zeiss LSM10 microscope equipped at 40× magnification was employed to acquire the images, and the fluorescent signal was quantified using the ImageJ 1.53a software [27].

### 2.6 Statistical Analysis

Statistical analyses were performed using the GraphPad Prism 9 software (GraphPad Software Inc., La Jolla, CA, USA) for all 15 participants. Evaluation of variance (ANOVA) followed by Tukey's post hoc test was conducted for comparisons between groups. All data are expressed as means ± standard deviations (SD). *p* < 0.05 was considered statistically significant in all cases.

## 3 Results

### 3.1 PM Exposure Promotes the Degradation of Extracellular Matrix Components

**FIGURE 1:**
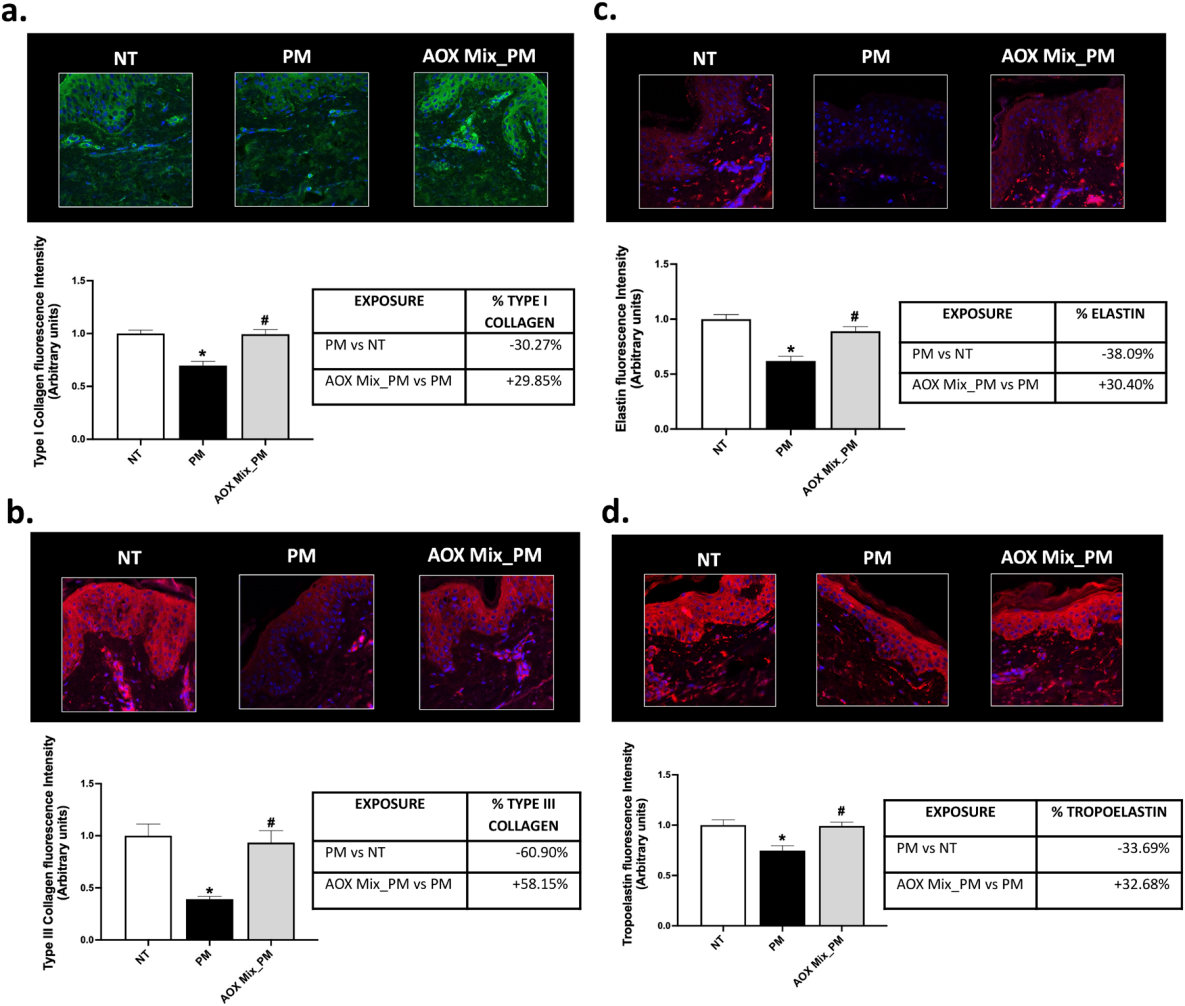
Expression of cutaneous ECM components. Immunofluorescence staining for type I collagen (a), type III collagen (b), elastin (c), and tropoelastin (d) in skin explants of subjects exposed to PM for 4 days and pre‐treated daily with AOX Mix. Green staining represents type I collagen, red staining represents type III collagen, elastin, and tropoelastin. Blue staining (DAPI) represents nuclei. The expression of the signal of each marker has been quantified using ImageJ software and graphed. Images were taken at 40× magnification and are representative of one donor. Data are the results of the averages of experiments conducted on 15 patients, *p < 0.05 PM exposure versus NT; ^#^ *p* < 0.05 AOX Mix pre‐treatment versus PM exposure by 1‐way ANOVA followed by Tukey's post hoc comparison test.

Structural proteins such as elastin and collagen are essential components of the extracellular matrix (ECM) in cutaneous tissue, providing skin elasticity and structural support [28, 29]. While the loss of these fibers is a natural part of the aging process, environmental insults may accelerate this process via the activation of inflammatory and oxidative pathways that promote premature ECM degradation [2]. In our study, we observed a 30%–40% decrease in the levels of type I collagen (Figure 1a), elastin, and the elastin monomer tropoelastin (Figure 1c,d) in skin biopsies from patients exposed to PM. Notably, the most significant reduction was observed in type III collagen (Figure 1b), with a decrease of nearly 60% in PM‐exposed skin compared to unexposed areas. In all cases, topical application of AOX Mix successfully prevented the PM‐induced loss of proteins related to ECM components.

### 3.2 Oxidative Stress and Inflammation

**FIGURE 2:**
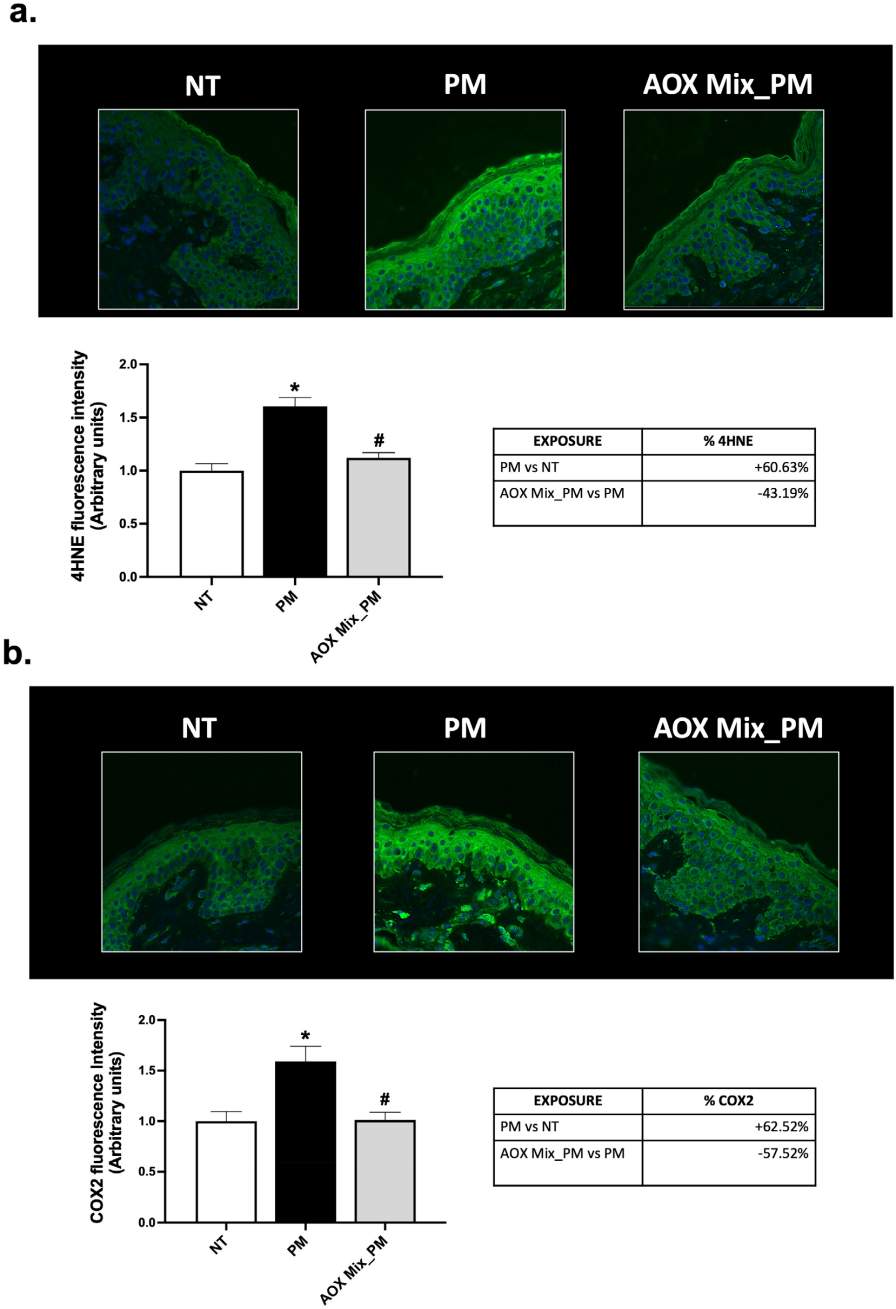
Oxidative stress and inflammation. Immunofluorescence staining for 4HNE (a) and COX2 (b) in skin explants of subjects exposed to PM for 4 days and pre‐treated daily with AOX Mix. Green staining represents both markers; blue staining (DAPI) represents nuclei. The expression of the signal of each marker has been quantified using ImageJ software and graphed. Images were taken at 40× magnification and are representative of one donor. Data are the results of the averages of experiments conducted on 15 patients, *p < 0.05 PM exposure versus NT; ^#^ *p* < 0.05 AOX Mix pre‐treatment versus PM exposure by 1‐way ANOVA followed by Tukey's post hoc comparison test.

Consistent with early signs of premature aging, our results indicate that PM exposure promotes the establishment of cutaneous oxidative and inflammatory responses. As shown in Figure 2, human skin exposed to PM displayed significantly increased levels of 4HNE (+60.63%) (Figure 2a), a reactive aldehyde produced by lipid peroxidative events, and of COX2 (+62.52%) (Figure 2b), the enzyme responsible for the biosynthesis of prostanoids [30]. Application of the AOX Mix prevented skin oxinflammatory damage induced by PM by restoring the basal levels of both 4HNE (−43%) and COX2 (−57%) compared to untreated PM‐exposed tissue.

### 3.3 PM Exposure Promotes Cutaneous Structural Damage

**FIGURE 3:**
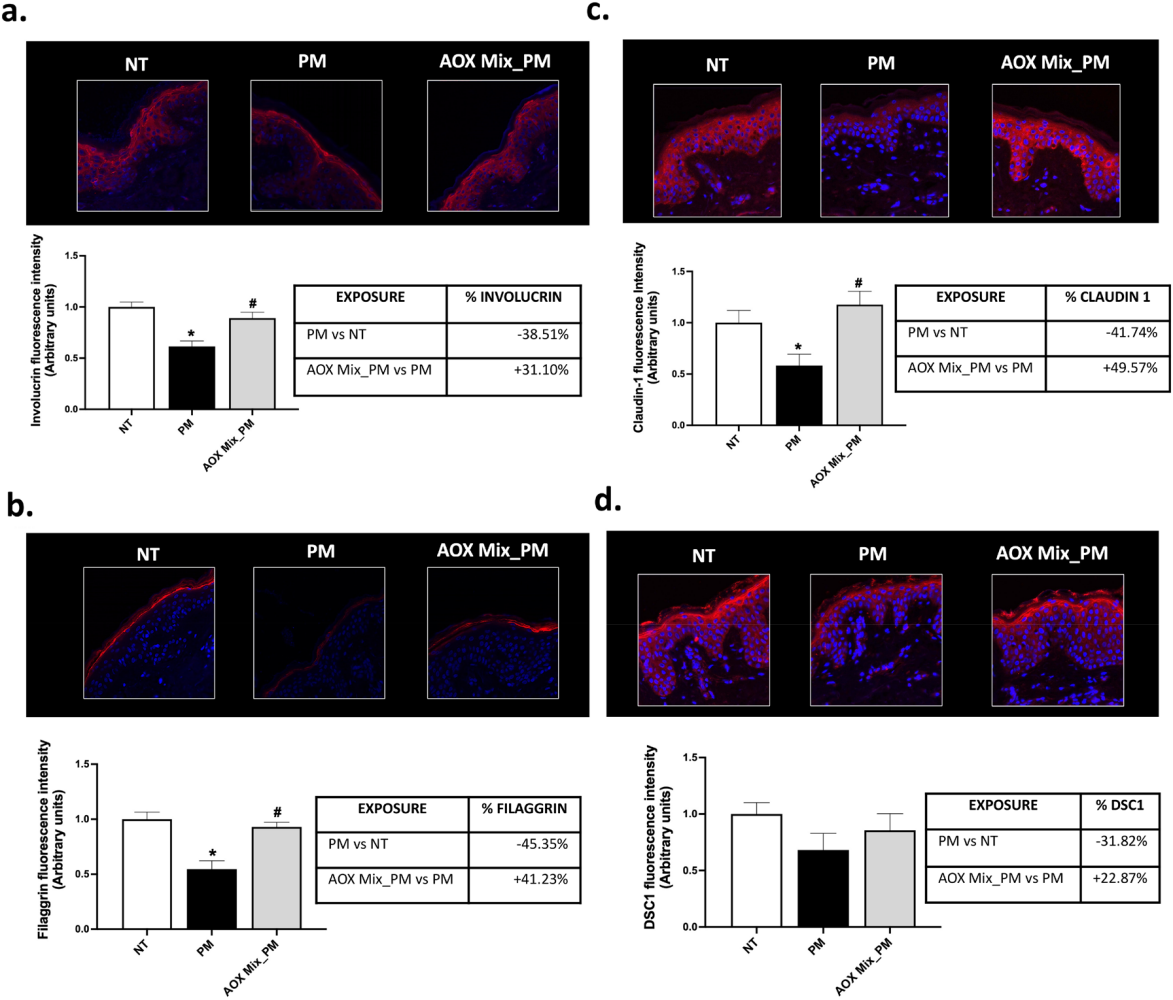
Cutaneous structural damage. Immunofluorescence staining for involucrin (a), filaggrin (b), claudin‐1 (c), and DSC1 (d) in skin explants of subjects exposed to PM for 4 days and pre‐treated daily with AOX Mix. All markers are stained in green; blue staining (DAPI) represents nuclei. The expression of the signal of each marker has been quantified using ImageJ software and graphed. Images were taken at 40× magnification and are representative of one donor. Data are the results of the averages of experiments conducted on 15 patients, *p < 0.05 PM exposure versus NT; ^#^ *p* < 0.05 AOX Mix pre‐treatment versus PM exposure by 1‐way ANOVA followed by Tukey's post hoc comparison test.

Oxinflammatory responses, together with the loss of skin elasticity and strength, may render the cutaneous tissue more prone to structural damage and thereby susceptible to environmental insults. Our findings show that PM exposure significantly impaired the expression of involucrin (Figure 3a) and filaggrin (Figure 3b)—two key proteins involved in skin differentiation and components of the cornified cell envelope of the skin [31, 32]. Importantly, the skin of subjects treated with AOX Mix preserved involucrin (+31%) and filaggrin (+41%) levels compared to PM exposed skin tissue.

Skin functionality is mainly ensured by the presence of intercellular adhesion complexes, such as tight junctions and desmosomes, which facilitate cellular communication by regulating the diffusion of molecules across tissues as well as by keeping cell–cell adhesion [33]. Exposure to PM downregulated the expression of claudin‐1 (−42%) (Figure 3c), an important component of tight junctions present within the skin, and to a lesser extent of DSC1 (−32%) (Figure 3d), one of the main components of desmosomes, compared to unexposed skin areas [33]. Topical AOX Mix application successfully preserved the levels of these two proteins. These results indicate that topical application of AOX Mix is effective in maintaining the integrity and functionality of the skin under PM exposure.

## 4 Discussion

Exposure to environmental insults has been associated with skin inflammaging, characterized by increased levels of pro‐inflammatory cytokines; these cytokines can activate a cellular cascade of events that ultimately trigger premature aging pathways [34]. Cellular responses to oxidative outdoor stressors involve the release of ROS, chemokines, inflammatory cytokines, and matrix metalloproteinases (MMPs), all of which contribute to cellular damage and, eventually, cell death [35]. Prolonged inflammation can promote increased expression of ROS production and degradation of components of the ECM, such as collagen and elastin, accelerating cutaneous aging [36].

PM_10_, in particular, has been found to trigger skin inflammation and redox signaling pathways, increasing levels of various inflammatory cytokines such as IL‐1β, IL‐6, IL‐8, IL‐33, as well as ROS and the activation matrix metalloproteinases (MMP‐1, MMP‐3) which degrade collagen type I and elastin [37]. The supplementation and application of natural antioxidant compounds offer a promising strategy to inhibit age‐related signaling pathways [18, 38]. In our study, the application of an antioxidant formulation (AOX Mix) containing ascorbic acid, ferulic acid, and tocopherol could prevent skin damage in subjects exposed to PM for 4 days.

Exposure of subjects to PM promoted decreased levels of ECM components such as type I collagen (Figure 1a), elastin, and its monomer tropoelastin (Figure 1c,d **)**, with a predisposition for type III collagen (Figure 1b) in skin samples. These ECM proteins are essential for maintaining the strength and elasticity of connective tissues. In particular, type I and III collagens are the most abundant fibrillar collagens in the skin, mainly produced by fibroblasts. Together they form a structural network responsible for mechanical stability. Type I collagen is predominantly responsible for tensile strength, generating thicker fibers, whereas type III collagen is more involved in the elastic rebound of the skin, and therefore involved in the early stages of wound healing [28] [39]. Exhibiting a more significant reduction compared to type I collagen (Figure 1a,b), type III collagen may be the earliest ECM protein affected by the detrimental effects of PM exposure. Type I and III collagen undergo different degradation processes, involving different MMPs such as MMP‐1‐2‐3 and − 9 [28]. Therefore, it is plausible that, under our experimental conditions, PM exposure induced type I and III collagen degradation, and in particular, the loss of type I collagen becomes even more evident at longer PM exposure [40].

Along with ECM degradation, we found increased levels of 4HNE (Figure 2a), one of the major lipid peroxidation products raised from the interaction between lipids and air pollutants [41], accompanied by increased levels of the inflammatory markers COX2 **(**Figure 2b **)**, whose upregulation was counteracted by AOX mix application. We previously demonstrated that exposure of human skin explants to air pollutants, including PM, ozone, and UV radiations, can promote the activation of the AhR receptor as well as other pathways of the inflammatory (NF‐kB, inflammasomes) leading to MMPs induction and collagen degradation [16, 26, 27, 42]. Also in this case, the application of antioxidant formulations helped prevent the skin damage [16, 43, 44]. In particular, the AhR receptor and p38 mitogen‐activated protein kinases (MAPK) pathways have been identified as the principal mediators of the oxinflammatory response of the skin to air pollutants exposure [45]. For instance, co‐culture experiments conducted on human dermal fibroblasts with human keratinocytes revealed that exposure to PM can trigger the release of inflammatory cytokines and COX2 via the activation of the AhR receptor/p38 MAPK that can lead to the activation of MMPs, thus promoting skin inflammaging [46]. Therefore, it is possible that also in this study, AhR might play a role in PM‐induced production of 4HNE and inflammatory status. The formation of an oxinflammatory environment is often linked to an increased risk of structural damage to the skin. A study demonstrated that subjects living in a high‐PM_2.5_ environment display decreased levels of skin structural proteins such as filaggrin, loricrin, and even desmosome constituents such as desmocollin‐1, correlated with increased levels of Ahr and inflammation [6]. Interestingly, TNF‐α triggered by PM exposure was found to downregulate filaggrin expression via the MAPK/c‐JNK pathway [6]. All these events are correlated with increased ROS production, able to trigger the activation of transcription factors and pathways such as AhR and NOTCH1 [15], while treatment with antioxidant molecules such as N‐Acetyl Cysteine can prevent skin damage [15]. Natural antioxidant compounds have been shown to reduce PM2.5‐induced intracellular ROS levels, apoptosis, and autophagy in human keratinocytes by modulating the MAPK pathway, thus protecting against the induction of MMPs and cell senescence [22, 47]. They can also protect against the structural skin damage promoted by PM and other air pollutants by restoring the expression levels of claudin‐1 and water channel aquaporin 3 (AQP3) [16, 48]. Consistent with these findings, our results showed that the skin of subjects pre‐treated with AOX Mix and exposed to PM showed significantly increased levels of structural proteins (Filaggrin and Involucrin) compared to PM‐exposed skin (Figure 3a,b). In addition, although less evident, PM exposure promoted a decrease in the expression levels of the tight junction claudin‐1 and the desmosome component DSC1 (Figure 3c,d), whose integrity favors cellular communication and strength to the skin tissues [49, 50]. Although not directly assessed, this might reflect an initial impairment of the skin barrier function that might be more evident with a prolonged exposure to PM. It is plausible that the experimental conditions adopted for this study primarily contribute to the oxinflammatory responses and the structural damage of the skin, whereas prolonged exposure might be necessary to appreciate a significant secondary effect of the oxinflammatory damage in terms of impairment of skin barrier function. Nonetheless, in all cases, the AOX Mix application could restore the levels of claudin‐1 and DSC1 at baseline, suggesting that the loss of these proteins is likely related to the PM‐induced oxinflammatory events. This supports the potential benefit of daily AOX Mix application in protecting the skin from long‐term pollution‐induced damage.

It is worth mentioning that the skin microbiome plays an important role in protecting the cutaneous tissue from pathogens and external insults [51]. Prolonged exposure to environmental pollutants promotes the alteration of skin microbiome response, rendering the skin more prone to the attack of other pathogens that may concur with the establishment of an oxinflammatory environment [52, 53]. For instance, many skin conditions such as atopic dermatitis display an altered skin barrier function, due to an impairment in tight junctions and structural protein expression, as well as an altered microbiome, all figures that contribute to an immunological impairment of the skin [54, 55]. Antimicrobial peptides (AMPs) such as LL‐37, human β‐defensins, and S100A7 play an important role in improving tight junction barrier function and preventing AhR activation, inhibiting the oxinflammatory response of the skin [56]. Therefore, although it was not measured in the present study, it is conceivable that an alteration of skin microbiota promoted by PM exposure, as suggested by others, might exacerbate pollution‐induced skin damage [57]. For instance, in a previous study, we were able to demonstrate that skin exposed to ozone, another noxious pollutant, could alter the cutaneous AMPs [52]. Since the application of antioxidant/moisturizing formulations and the use of vitamins A, B3, C, D, and E have been found efficient in preserving skin microbiota [58, 59], the daily treatment with AOX Mix might have helped in regulating the skin microbiota, ensuring its protective activity against PM exposure, although this aspect was not evaluated in the present study.

In conclusion, this study demonstrates that exposure to PM disrupts key proteins responsible for maintaining the skin's structural integrity and barrier function, primarily through the activation of oxinflammatory pathways. Topical application of the AOX Mix mitigated oxidative stress and inflammatory responses, preserving important components of the ECM and structural proteins of the cutaneous tissues, counteracting premature skin aging. Although the limited sample size constrains the generalizability of our findings to a global population, this work lays a valuable foundation for future larger‐scale research. Regular use of antioxidant‐based cosmeceuticals is a promising strategy to prevent the detrimental effect of environmental pollutants.

## Conflicts of Interest

Xi Yan, Stephen Lynch, Sara Anderias, are L'Oréal Research and Innovation, New York, NY, USA employees. Hina Choudhary and Stacy White are SkinCeuticals employees, New York, NY, USA.

## Data Availability

The data that support the findings of this study are available on request from the corresponding author. The data are not publicly available due to privacy or ethical restrictions.
